# The Elements of Modern Domestic Medicine

**Published:** 1887-11

**Authors:** 


The Elements of Modern Domestic Medicine. By Henry G. Hanchett, M. D., revised by A. H. Laidlaw, A. M., M. D. New York: Charles T. Hurlburt, 1887.

This is a most excellent family book, containing clear directions for simple treatment of the members of a family in emergencies, when a physician is not at hand. We have rarely met with any book devoted to this purpose in which the style is so clear, concise, and convincing. The remedies, apart from the homœopathic medicines, are all of the common-sense order.

We regret to find this otherwise excellent book marred by recommending the administration of some remedies after the manner of the eclectics. However, this is not of so much consequence in a book of domestic practice, as the measures it advocates are for the most part only for use "till the doctor comes."

One of the most interesting and valuable portions of this book is that relating to "Sexual Health." We can heartily commend it. As the subjects of which it treats are necessarily of such a nature that but few persons among the laity, and they the heads of families, should read anything concerning them, the author has very wisely not incorporated them in the general text of the book, where they would be certain to meet the eyes of the young sons and daughters of the family. They are incorporated in a separate volume that can be secreted from the inquiring gaze of the young people. This in itself is no inconsiderable merit. W. M. J.

